# Regression of a Non-Irradiated Lung Adenocarcinoma During Glioblastoma-Directed Chemoradiotherapy: A Case Report

**DOI:** 10.3390/curroncol33040188

**Published:** 2026-03-28

**Authors:** Mizuki Iwanaga, Yosuke Dotsu, Takeshi Hiu, Nozomi Ueki, Yudai Hirano, Takatomo Tokito, Toru Morikawa, Seiya Kaneko, Noritaka Honda, Kazumasa Akagi, Hiromi Tomono, Midori Matsuo, Hirokazu Taniguchi, Shinnosuke Takemoto, Shinji Okano, Hiroshi Mukae

**Affiliations:** 1 Department of Respiratory Medicine, Nagasaki University Graduate School of Biomedical Sciences, Nagasaki 852-8501, Japan; iwami11@nagasaki-u.ac.jp (M.I.); yhirano@nagasaki-u.ac.jp (Y.H.); t-mori@nagasaki-u.ac.jp (T.M.); s-kaneko36@nagasaki-u.ac.jp (S.K.); nhonda@nagasaki-u.ac.jp (N.H.); htomono@nagasaki-u.ac.jp (H.T.); shinnosuke-takemoto@nagasaki-u.ac.jp (S.T.); hmukae@nagasaki-u.ac.jp (H.M.); 2 Department of Neurosurgery, Nagasaki University Graduate School of Biomedical Sciences, Nagasaki 852-8501, Japan; takeshihiu@nagasaki-u.ac.jp; 3 Department of Tumor and Diagnostic Pathology, Atomic Bomb Disease Institute, Nagasaki University, Nagasaki 852-8501, Japan; nozomiueki@nagasaki-u.ac.jp; 4 Department of Respiratory Medicine, Sasebo City General Hospital, Sasebo 857-8511, Japan; t-takatomo@hospital.sasebo.nagasaki.jp; 5 Clinical Oncology Center, Nagasaki University Hospital, Nagasaki 852-8501, Japan; kaakagi@nagasaki-u.ac.jp (K.A.); hirokazu_pc@nagasaki-u.ac.jp (H.T.); 6 Clinical Research Center, Nagasaki University Hospital, Nagasaki 852-8501, Japan; mi-shimada@nagasaki-u.ac.jp; 7 Department of Pathology, Nagasaki University Graduate School of Biomedical Sciences, Nagasaki 852-8501, Japan; okanos@nagasaki-u.ac.jp

**Keywords:** glioblastoma, lung cancer, chemoradiotherapy, case report, immunogenic cell death, cross-organ tumor regression

## Abstract

Treatment-associated regression of tumors outside the irradiated field has occasionally been reported, but the underlying mechanisms remain unclear, particularly in the context of central nervous system (CNS)–directed therapy. Glioblastoma (GBM) is commonly treated with radiotherapy and temozolomide, both of which may influence tumor biology and the systemic environment. We report a patient with synchronous primary GBM and early-stage lung adenocarcinoma who underwent craniotomy followed by intensity-modulated radiotherapy with concurrent temozolomide for GBM. During GBM-directed chemoradiotherapy, the untreated pulmonary lesion demonstrated progressive regression without any lung-specific therapy, temporally coinciding with CNS-targeted treatment. Although comprehensive immunophenotyping was not feasible, longitudinal changes in the proportion of peripheral blood lymphocytes were observed during therapy. These findings represent a clinical observation characterized by a temporal association between CNS-directed treatment and regression of a distant, non-irradiated tumor. However, the underlying mechanism remains uncertain, and a contribution from systemic temozolomide exposure cannot be excluded. While treatment-related systemic effects may be considered, no specific causal mechanism can be established based on this single case. This case highlights an unusual clinical observation that may warrant further investigation. Further studies are needed to clarify the relationship between CNS-directed therapies and systemic tumor behavior.

## Simple Summary

Radiotherapy and chemotherapy have occasionally been associated with regression of tumors outside the irradiated field. We report a patient with synchronous glioblastoma and lung adenocarcinoma who experienced shrinkage of an untreated lung lesion during glioblastoma-directed chemoradiotherapy, without any lung-specific treatment. This regression occurred in parallel with changes in the proportion of peripheral blood lymphocytes. Although based on a single case, this observation describes a temporal association between glioblastoma-directed therapy and regression of a distant, non-irradiated tumor. However, the underlying mechanism remains uncertain, and a contribution from systemic treatment effects, including temozolomide, cannot be excluded. Further investigation is needed to clarify whether therapies targeting the central nervous system may be associated with changes in systemic tumor behavior.

## 1. Introduction

Treatment-induced systemic antitumor immunity and abscopal-like responses remain incompletely understood in solid tumors, particularly in the context of central nervous system (CNS)-directed therapy. Spontaneous regression (SR) of malignant tumors, defined as partial or complete tumor disappearance without specific antitumor treatment, is an exceptionally rare phenomenon [1]. Although SR has been reported in malignancies such as renal cell carcinoma, melanoma, and neuroblastoma [2], it is extremely uncommon in lung cancer [3].

Glioblastoma (GBM), one of the most aggressive primary brain tumors, is typically treated with a combination of radiotherapy and temozolomide, both of which can induce immunogenic cell death (ICD) and modulate systemic immune activation [4]. Despite increasing recognition of radiation- and chemotherapy-induced immune modulation, little is known about how GBM-directed therapy reshapes peripheral immunity or whether such immune activation can influence synchronous extracranial malignancies. This knowledge gap is particularly relevant to understanding abscopal-like phenomena in non-irradiated organs.

Here, we describe a patient with synchronous GBM and lung adenocarcinoma who exhibited regression of the non-irradiated pulmonary lesion during GBM-directed chemoradiotherapy. This translational clinical observation describes a temporal association between GBM-directed chemoradiotherapy and regression of a synchronous, non-irradiated lung adenocarcinoma, with an uncertain underlying mechanism.

## 2. Case Presentation

### 2.1. Patient and Ethical Approval

A 75-year-old Japanese woman with no smoking history was evaluated at our institution. Written informed consent was obtained from the patient for publication of clinical data and images, and the study was approved by the Institutional Review Board of Nagasaki University Hospital (Approval number: 25111309).

### 2.2. Imaging and Pathological Evaluation

**Figure 1:**
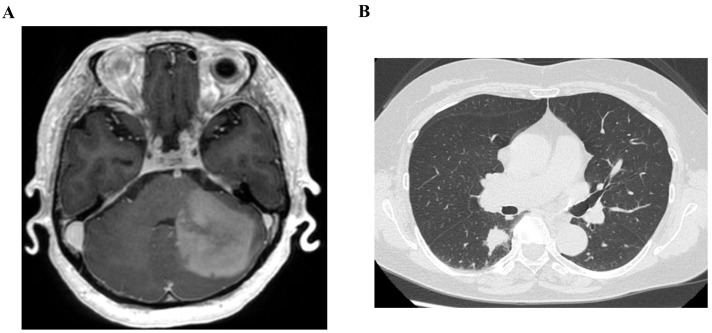
Radiologic features of synchronous glioblastoma and lung adenocarcinoma (**A**) Contrast-enhanced magnetic resonance imaging of the brain reveals a tumor lesion extending from the left cerebellar hemisphere to the medulla oblongata. (**B**) Chest computed tomography showing a spiculated nodule in the right lower lobe of the lung.

Contrast-enhanced magnetic resonance imaging (MRI) of the brain revealed a 47-mm mass extending from the left cerebellar hemisphere to the medulla oblongata (Figure 1A). Whole-body computed tomography (CT) demonstrated an 18-mm spiculated pulmonary nodule in the right lower lobe (Figure 1B). Craniotomy for diagnostic biopsy and bronchoscopy were performed to establish definitive diagnoses.

**Figure 2:**
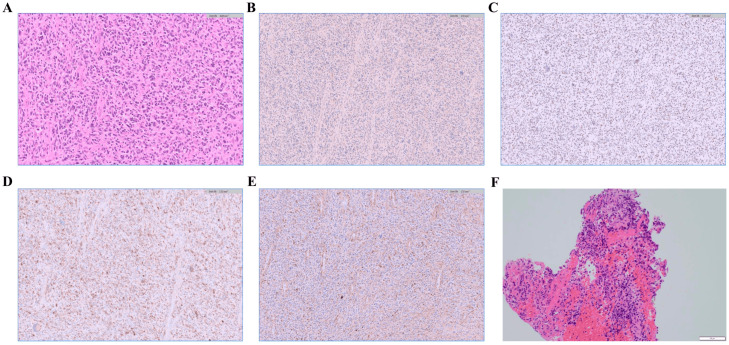
Histological confirmation of double primary malignancies. (**A**) Histological examination of the brain tumor obtained by craniotomy for biopsy reveals proliferation of atypical cells with pleomorphic, large, and bizarre nuclei. Although necrosis and mitosis are not prominent, microvascular proliferation is evident (Hematoxylin and eosin stain at ×20 magnification). (**B**) Immunohistochemistry for IDH1-R132H is negative, supporting an IDH-wild-type phenotype (at ×10 magnification). (**C**) ATRX expression is retained, indicating no loss of nuclear expression (at ×10 magnification). (**D**) p53 demonstrates strong overexpression, consistent with aberrant accumulation (at ×10 magnification). (**E**) GFAP is focally positive, highlighting focal glial differentiation within the lesion (at ×10 magnification). (**F**) The transbronchial lung biopsy specimen showed polygonal cancer cells proliferating in a lepidic growth pattern, consistent with adenocarcinoma (Hematoxylin and eosin stain at ×200 magnification).

Histopathological examination confirmed synchronous double primary malignancies: GBM in the brain and lung adenocarcinoma (Figure 2A–F). The lung cancer was staged as IA, whereas the brain tumor was classified as Central Nervous System (CNS) World Health Organization grade 4.

### 2.3. Treatment Protocol

Given the aggressive nature of the GBM, CNS-directed therapy was prioritized. The patient underwent maximum safe surgical resection of the contrast-enhancing tumor followed by concurrent temozolomide and intensity-modulated radiotherapy (total dose 40.05 Gy in 15 fractions), consistent with standard hypofractionated regimens for older patients with GBM according to the STUPP protocol. Concurrent temozolomide was administered at 75 mg/m^2^ daily during radiotherapy, followed by adjuvant temozolomide at 150 mg/m^2^ for 5 days every 28 days, with subsequent dose escalation to 200 mg/m^2^.

Following an initial radiologic response, a new contrast-enhancing lesion at the left cerebellar peduncle was detected four weeks after the completion of radiotherapy. Additional imaging evaluation, including perfusion MRI and magnetic resonance spectroscopic imaging (MRSI), was performed. The contrast-enhancing ring-shaped lesion corresponded to an area demonstrating an elevated choline peak on MRSI, a finding generally considered suggestive of tumor recurrence rather than treatment-related pseudoprogression. Although pseudoprogression cannot be completely excluded, the overall imaging findings favored tumor recurrence. Based on this assessment, bevacizumab (10 mg/kg) was added to ongoing temozolomide therapy and has been continued as part of the patient’s ongoing treatment with regular radiologic follow-up.

Molecular testing for EGFR mutation and ALK rearrangement was not performed because the lung adenocarcinoma was clinically staged as early-stage disease and surgical resection was planned as definitive therapy. Similarly, the MGMT promoter methylation status was not assessed because the treatment strategy for glioblastoma had already been determined based on the patient’s clinical condition.

### 2.4. Peripheral Blood Analysis

Longitudinal peripheral blood counts were obtained during treatment. Because the patient exhibited marked leukocytosis suspected to be related to granulocyte colony-stimulating factor–producing tumor activity, lymphocyte proportion rather than absolute lymphocyte count was used as a surrogate marker of systemic immune dynamics. Serum granulocyte colony-stimulating factor (G-CSF) levels were not measured; however, the marked neutrophil-predominant leukocytosis in the absence of infection supported a clinical suspicion of G-CSF–producing tumor activity.

### 2.5. Clinical Course and Radiologic Response

**Figure 3:**
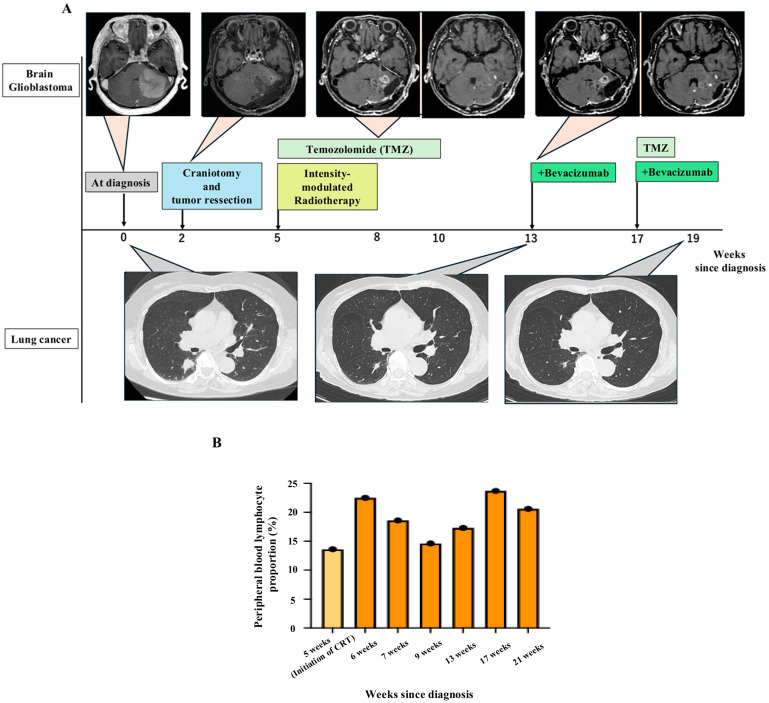
Clinical course and systemic immune dynamics during glioblastoma-directed chemoradiotherapy. (**A**) Clinical timeline of central nervous system-directed treatment and radiologic response of synchronous glioblastoma and lung adenocarcinoma. Serial brain MRI images showing the clinical course of glioblastoma from diagnosis through chemoradiotherapy and subsequent bevacizumab treatment at weeks 0, 2, 8, and 13. Corresponding chest computed tomography images demonstrating progressive reduction in the untreated pulmonary lesion at weeks 0, 13, and 19 after diagnosis. (**B**) Longitudinal analysis of peripheral blood lymphocyte proportion during treatment, used as a surrogate marker of systemic immune dynamics. Abbreviations: CRT, chemoradiotherapy.

Serial chest CT performed for lung cancer surveillance demonstrated progressive regression of the non-irradiated right lower lobe pulmonary nodule during GBM-directed chemoradiotherapy, with the longest diameter decreasing from 18 mm at diagnosis to 12 mm at week 13 (Figure 3A). This initial reduction occurred prior to the initiation of bevacizumab therapy. Further shrinkage to 9 mm at week 19 was observed 6 weeks after the initiation of combined temozolomide and bevacizumab therapy, despite the absence of lung cancer–directed treatment.

### 2.6. Longitudinal Changes in Peripheral Lymphocyte Proportion

Concomitant with tumor regression, peripheral blood analysis revealed an upward trend in lymphocyte proportion relative to pretreatment levels (Figure 3B), temporally associated with CNS-targeted therapy. Although comprehensive immunophenotyping was not feasible, these longitudinal changes may be consistent with treatment-induced systemic immune activation.

### 2.7. Clinical Outcome

To date, the patient remains alive without any specific therapy for lung adenocarcinoma and continues to show no evidence of disease progression. The overall clinical timeline is summarized in Figure 3A,B.

## 3. Discussion

Non-small cell lung cancer (NSCLC) is the most prevalent form of lung cancer, with brain metastases occurring in approximately 12.6–22.6% of patients with advanced disease [5]. Most metastatic brain tumors originating from extracranial primaries arise from lung cancer [5], and synchronous double primary malignancies involving both GBM and lung cancer have been rarely reported [6,7,8]. Beyond its rarity, the present clinical observation highlights regression of a non-irradiated lung adenocarcinoma during GBM-directed chemoradiotherapy, representing an unusual clinical observation with an uncertain underlying mechanism rather than evidence of a defined biological effect.

Cases in which treatment of one malignancy induces regression of another are summarized in Table 1 [9,10,11,12,13,14,15,16,17,18,19,20,21,22]. Radiotherapy to the CNS may modulate systemic immunity through multiple interconnected mechanisms. One plausible explanation is the abscopal effect, whereby localized irradiation triggers regression of distant, non-irradiated tumors via immune-mediated pathways. This phenomenon is thought to result from radiation-induced release of tumor-associated antigens and subsequent activation of systemic antitumor immunity, particularly cytotoxic T-cell responses [4]. In the present case, the temporal association between brain irradiation and regression of the pulmonary lesion may be consistent with treatment-associated systemic effects. However, a causal relationship cannot be established.

Consistent with this hypothesis, longitudinal analysis revealed an upward trend in peripheral blood lymphocyte proportion following the initiation of chemoradiotherapy, potentially reflecting treatment-associated immune modulation. Because the patient exhibited marked pretreatment leukocytosis suspected to be related to G-CSF–producing tumor activity, lymphocyte proportion rather than absolute lymphocyte count was evaluated to avoid confounding by excessive neutrophilia. Peripheral lymphocyte proportion is a nonspecific indicator and cannot be considered direct evidence of systemic immune activation, particularly in the context of surgery, radiotherapy, and ongoing systemic therapy. Although this surrogate approach has inherent limitations, the temporal increase in lymphocyte proportion may still be consistent with treatment-associated immune modulation.

The combination of radiotherapy and immune checkpoint inhibitors has been shown to exert synergistic antitumor effects through the enhancement of ICD. Radiation induces tumor cell apoptosis and necrosis, accompanied by the release of danger-associated molecular patterns (DAMPs), including calreticulin, HMGB1, and ATP, which promote dendritic cell maturation and subsequent T-cell activation [23]. Immune checkpoint blockade further amplifies these responses by relieving inhibitory signaling within the tumor microenvironment, thereby enhancing systemic antitumor immunity [24]. Although abscopal responses have been reported in cases combining radiotherapy with immune checkpoint inhibitors, such effects have not been established for temozolomide alone.

Alkylating agents such as temozolomide primarily exert their effects through DNA damage–induced apoptosis and cellular senescence. Radiotherapy similarly causes extensive DNA damage, leading to DAMP release and the initiation of dendritic cell maturation and antigen presentation, ultimately activating cytotoxic T cells whose antitumor activity may be further enhanced by radiotherapy [25]. In addition, temozolomide may modulate the tumor immune microenvironment by inducing cytokines such as interleukin-6 and interleukin-8 via activation of the NF-κB signaling pathway [26].

An alternative explanation is that systemic temozolomide exposure may have contributed to the regression of the untreated lung adenocarcinoma. Because temozolomide was administered systemically and no direct assessment of tumor sensitivity was available, this possibility cannot be excluded. In addition, MGMT promoter methylation status was not assessed in this case; therefore, potential sensitivity to temozolomide cannot be evaluated.

However, temozolomide does not have an established therapeutic role in lung adenocarcinoma or NSCLC, and prior clinical studies have demonstrated limited and inconsistent antitumor activity in this setting [27]. Furthermore, available studies have largely focused on patients with brain metastases rather than untreated extracranial primary lesions. Therefore, while temozolomide may have contributed to the observed tumor regression, the available clinical evidence does not support a consistent or clinically meaningful antitumor effect in this disease context, and its relative contribution cannot be determined in this single case.

Furthermore, no prospective randomized trials have demonstrated meaningful antitumor efficacy of bevacizumab monotherapy as first-line treatment for advanced NSCLC. Consistent with this, bevacizumab predominantly mediates antivascular and anti-angiogenic effects, including the modulation of tumor vasculature and permeability, rather than direct tumor cytotoxicity [28,29]. Therefore, although the potential contribution of these agents cannot be completely excluded, their known pharmacologic profiles make them less likely to fully account for the observed regression of the untreated pulmonary lesion in this single case. However, alternative explanations for the observed regression should also be considered. In particular, the relatively indolent biological behavior of early-stage lung adenocarcinoma and the potential variability in radiologic measurement may have contributed to the apparent reduction in measured tumor size [30].

Given that bevacizumab can reduce contrast enhancement through its antiangiogenic effects, radiologic pseudoresponse should be considered while interpreting follow-up imaging. In the present case, radiologic assessment was performed not only using contrast-enhanced sequences but also with careful evaluation of FLAIR image and the overall clinical course to minimize misinterpretation related to anti-angiogenic therapy.

Although incidental spontaneous regression has been reported in lung cancer [31], this phenomenon remains exceedingly rare [3], and no single mechanism can be definitively established in the present case. This study is limited by its single-patient nature, lack of comprehensive immunophenotyping, and relatively short observation period. In addition, the molecular status of EGFR, ALK, and MGMT was not available because treatment strategies had already been determined based on clinical considerations.

Nevertheless, the magnitude and temporal precision of the systemic response provide an intriguing clinical observation with an uncertain underlying mechanism. Further investigation is warranted to clarify the relationship between GBM-directed therapy and systemic tumor behavior.

In summary, this translational clinical observation describes a temporal association between GBM-directed chemoradiotherapy and regression of a distant, non-irradiated tumor. However, the underlying mechanism remains uncertain, and multiple explanations—including systemic treatment effects—should be considered.

## 4. Conclusions

This study describes a rare clinical observation of regression of a synchronous, non-irradiated lung adenocarcinoma during GBM-directed chemoradiotherapy. However, the underlying mechanism remains uncertain, and a contribution from systemic temozolomide exposure cannot be excluded. Rather than demonstrating a specific biological mechanism, this case highlights a temporal association that may warrant further investigation. Future prospective studies incorporating detailed immune monitoring and molecular characterization will be necessary to clarify the relationship between CNS-directed therapy and systemic tumor behavior.

## Figures and Tables

**Table 1:** Cases of spontaneous tumor regression in one malignancy following treatment of another primary cancer.

Author	Year	Cancer Combination	Treatment Site	Modality	Regression Observed in
Kim JO, et al. [9]	2019	Cholangiocarcinoma and NSCLC	Lung	SBRT	Hepatic metastasis of cholangiocarcinoma
Chino F, et al. [10]	2018	Hepatocellular carcinoma and NSCLC	Lung	SBRT	Hepatocellular carcinoma
Ebner DK, et al. [11]	2017	colorectal cancer	Aortic lymph node	Radiation therapy	Subclavian lymph node
Cong Y, et al. [12]	2016	NSCLC	Primary lung lesion	SABR	Lung Metastasis
Desar IME, et al. [13]	2016	Diffuse-type giant cell tumor	Primary lung lesion	Radiation therapy	Lung metastasis and mediastinal lymph node
Joe MB, et al. [14]	2017	Squamous cell carcinoma of the anal canal	Pelvis	Radiation therapy	Anal, bone and liver
Okuma K, et al. [15]	2011	Hepatocellular carcinoma	Mediastinal lymph node	Radiation therapy	Lung metastasis
Hamilton AJ, et al. [16]	2018	NSCLC	brain	Radiation therapy	Lung
Siva S, et al. [17]	2013	NSCLC	Lung	SABR	Adrenal and bone metastasis
Golden EB, et al. [18]	2013	NSCLC	Liver	Radiation therapy + anti- CTLA-4	Lung, sacral and hilar/mediastinal lymph node
Postow MA, et al. [19]	2012	Melanoma	Paraspinal lesion	Radiation therapy + anti-CTLA-4	Hilar lymph node and spleen
Okwan-Duodu D, et al. [20]	2015	Melanoma	Brain	WBRT + IL-2	Lung and groin
Lin X, et al. [21]	2019	Lung adenocarcinoma	Brain	WBRT + anti-PD-L1	Lung
Parisi S, et al. [22]	2022	Lung adenocarcinoma	Brain	SRS + anti-PD-1	Lung and bone

NSCLC: Non-small cell lung cancer, SBRT: Stereotactic body radiotherapy, SABR: Stereotactic ablative radiation therapy, WBRT: Whole brain radiotherapy, SRS: Stereotactic radiosurgery.

## Data Availability

The original contributions presented in this study are included in the article. Further inquiries can be directed to the corresponding author.
